# The Effect of Mesenchymal Stem Cells, Adipose Tissue Derived Stem Cells, and Cellular Stromal Vascular Fraction on the Repair of Acute Anal Sphincter Injury in Rats

**DOI:** 10.3390/bioengineering9070318

**Published:** 2022-07-15

**Authors:** Wenbin Chen, Zijian He, Shuyu Li, Zixin Wu, Jin Tan, Weifeng Yang, Guanwei Li, Xiaoting Pan, Yuying Liu, Feng-Juan Lyu, Wanglin Li

**Affiliations:** 1 Department of Colorectal and Anal Surgery, The Second Affiliated Hospital, School of Medicine, South China University of Technology, Guangzhou 510641, China; mcchenwb@mail.scut.edu.cn (W.C.); 201920153709@mail.scut.edu.cn (Z.H.); agnesng@foxmail.com (Z.W.); yangweifeng202011@163.com (W.Y.); eyliguanwei@scut.edu.cn (G.L.); 2 School of Biology and Biological Engineering, South China University of Technology, Guangzhou 510641, China; shuyu_li2001@163.com; 3 Joint Center for Regenerative Medicine Research of South China University of Technology and The University of Western Australia, School of Medicine, South China University of Technology, Guangzhou 510641, China; 202020156134@mail.scut.edu.cn (J.T.); mc202120157352@mail.scut.edu.cn (X.P.); mc202120157344@mail.scut.edu.cn (Y.L.)

**Keywords:** ASI, MSC, ADSC, CSVF, anal sphincter incontinence

## Abstract

Background: Anal sphincter incontinence (ASI) can cause a serious decline in the quality of life and can cause a socioeconomic burden. Studies have shown that bone marrow mesenchymal stem cells (MSC) have significant therapeutic effects on ASI, but the cost and risk of MSC harvest limit their further application. In contrast, adipose tissue derived stem cells (ADSC) and cellular stromal vascular fraction (CSVF) as stem cell sources have multipotency and the advantage of easy harvest. Objective: Here we aim to investigate the effects of ADSC and CSVF on treating ASI and compare them to that of bone marrow MSC. Methods: Bone marrow MSC, ADSC, and CSVF were obtained and labeled with green fluorescent protein (GFP), and CSVF was labeled with DIL. Sprague Dawley (SD) rats were divided into 5 groups. Four groups were injected with 0.2 mL phosphate buffer saline (PBS), 1 × 10^7^/0.2 mL of MSC, ADSC, or CSVF, respectively, after model establishment. The control group received no treatment. The repair was assessed by anal functional tests and immunostaining on day 5 and day 10 after injection. Results: MSC, ADSC, and CSVF significantly promoted tissue repair and the recovery of muscle contraction and electromyographic activity in ASI. The generation of myosatellite cells by injected MSC, ADSC, and CSVF was found in the wounded area. On day 5, CSVF showed highest therapeutic effect, while on day 10, MSC and ADSC showed higher therapeutic effects than CSVF. When comparing the effects of MSC and ADSC, ADSC was slightly better than MSC in the indexes of anal pressure, etc. Conclusion: ADSC and CVSF are alternative stem cell sources for ASI repair.

## 1. Introduction

Anal sphincter incontinence (ASI) is defined by an involuntary loss of gases, solids and/or liquid materials [1] through the anus. Although ASI is a benign disease, its impact on individuals and society cannot be ignored. Patients with ASI have a severely reduced quality of life and are prone to a range of psychosocial problems [2]. Dysfunction of the internal anal sphincter (ISA) and external anal sphincter (ESA) is an important pathophysiological mechanism leading to ASI. In particular, ASI can be caused by obstetric trauma during childbirth in women [3], and the anal sphincter dysfunction can be caused by episiotomy procedure [4]. The current treatment for sphincter injury is mainly conservative, such as dietary modification, antidiarrheal medication, and biofeedback, etc. Sphincter repair is often performed for larger deficient ISA or ESA. However, the efficacy of anal sphincter repair has been inconsistent, with poor long-term outcomes and poor efficacy in IAS. Stem cells, with the potential to differentiate into IAS or EAS as well as to produce anabolic cytokines, may provide a cure for ASI [5].

With the development of regenerative medicine and medical needs due to poor surgical outcomes, cell therapies have been widely studied for muscle repair. A cure for ASI is theoretically possible with the aid of stem cells.

Mesenchymal stem cells (MSC) [6] have the capacity to differentiate into cells of a mesenchymal lineage [7,8,9]. MSC can be derived from various sources [10] with differences in marker expression profile and differentiation potential [11]. To date, MSC [12,13,14], adipose-derived stem cells (ADSC) [15], and skeletal muscle-derived stem cells [16] have been used in preclinical and clinical studies over the past few years [17,18,19] and have shown significant therapeutic effects in treating ASI. Williams et al. [20] treated intrinsic urinary sphincter deficiency (ISD) with skeletal muscle precursor stem cells and showed improvement in muscle proportions and urethral function, and the local injection group showed better efficacy than the intravenous injection group. Mazzanti et al. [21] found that MSC promoted postoperative esophageal sphincter repair. Although bone marrow MSC has shown good therapeutic effects, bone marrow aspiration is an invasive procedure with risks and requires laboratory culture for expansion, which is time-consuming and expensive. In contrast, ADSC is derived from adipose tissue, which is simple to obtain, widely available, and has good efficacy in repairing injured anal sphincters [15]. Studies have been performed on the repair of anal sphincter injury by MSC and ADSC [19].

We are also interested in stromal vascular fraction (SVF), which contains ADSC (less than 10%) as well as the cellular and molecular microenvironmental components of ADSC [22], including mature adipocytes, fibroblasts, pericytes, macrophages, and blood cells, endothelial progenitor cells, etc. [23,24]. SVF also has multidirectional differentiation potential and immunomodulatory effect [25]. Among them, adipose stem cells and others can participate in regenerative processes, while endothelial cells can secrete an insulin-like growth factor. Endothelial cells can secrete an insulin-like growth factor (IGF-1) and a vascular endothelial growth factor (VEGF) [26]; macrophages and monocytes can also participate in the immune regulation process [27]. Taken together, these microenvironmental components are conducive to the survival and therapeutic effect of ADSC in vivo. Compared to ADSC, the preparation of SVF is even simpler, with single protease digestion yielding 2–6 × 10^6^ SVF cells (CSVF) per 1 mL of fat with ≥90% cell viability. Many studies have shown that ADSC and CSVF can promote tissue repairs, such as skeletal tissue [28,29,30,31], muscle tissue [32,33,34], nerve damage [35,36,37,38], and cardiovascular injury [39,40,41]. Meanwhile, the tissue source of ADSC and CSVF is widely available, and a large amount of adipose tissue can be obtained for ADSC and CSVF extraction during liposuction, manual liposuction, etc., while the invasive procedure in obtaining bone marrow MSC and muscle-derived stem cells have limited their application in clinic.

It is yet unclear whether ADSC can deliver a similar or higher therapeutic effect compared to MSC in treating ASI. Moreover, in the literature, CSVF has not been investigated for its potential in treating ASI. In this study, we investigated the therapeutic effects of MSC, ADSC, and CSVF on ASI repair in rat models and made comparison. Functional repair, including muscle contraction and electromyographic activity, histological appearance, and the survival, proliferation, and differentiation of implanted MSC, ADSC, and CSVF were investigated, with an aim to evaluate the potential of ADSC and CSVF in treating ASI.

## 2. Materials and Methods

### 2.1. Acquisition of MSC, ADSC, and SVF

MSC and ADSC were purchased from Cyagen at Passage 1, cultured in Sprague Dawley (SD) rat ADSC or MSC special cell culture medium (Cyagen) and expanded to Passage 4. For the preparation of CSVF, adipose tissue was cut into a paste, transferred into a 50 mL centrifuge tube, mixed with an equal amount of 0.25% type I collagenase, digested in a water bath shaker for 40 min at 37 °C, mixed with an equal amount of medium, and filtered through a 70 μm membrane. The filtrate was centrifuged at 1500 rpm for 5 min, resuspended in 1 mL of medium, incubated with 4 mL of erythrocyte lysate for 5 min, followed by centrifugation. The cell number was counted after trypan blue exclusion of dead cells under a phase-contrast microscope.

### 2.2. Characterization of MSC and ADSC

All induction solutions were purchased from Cyagen.

(1) Adipogenic induction and characterization: ADSC or MSC at Passage 4 were inoculated in 6-well plates at a density of 2 × 10^4^ cells per well. When the cell density reached 100%, the cells were subjected to four rounds of 2 mL of solution A (basal medium, fetal bovine serum, penicillin-streptomycin, giltamine, insulin, rosiglitazone, dexamethasone, 3-Isobutyl-1-methylxanthine) for three days and 2 mL of solution B (basal medium, fetal bovine serum, penicillin-streptomycin, giltamine, insulin) for 24 h. The culture was then maintained with B solution for 5 days. For characterization of adipogenesis, the plates were washed twice with phosphate buffer saline (PBS), fixed with 4% paraformaldehyde, stained with Oil Red O for 30 min, and washed with PBS for four times. The culture plates were then observed under a light microscope (Leica, DMIL LED, Heidelberg, Germany) to observe the effect of lipogenic staining.

(2) Chondrogenic induction and characterization: 4 × 10^5^ ADSC or MSC at Passage 4 were transferred into a 15 mL centrifuge tube and centrifuged at 250× *g* for 4 min. Then the supernatant was aspirated, and 0.05 mL premix was added to resuspend the precipitate, followed by centrifugation at 150× *g* for 5 min at room temperature. The precipitate was resuspended with 0.05 mL chondrogenic differentiation complete medium and centrifuged at 150× *g* for 5 min at room temperature. Then the cap of the tube was kept half-loose and incubated overnight in a 37 °C incubator with 5% CO_2_. When the cells started to appear clustered, the bottom of the centrifuge tube was gently flicked to make the cell spheres detach from the bottom of the tube. The medium was refreshed every 3 days, and after 25 days of induction, the spheres were fixed with 4% paraformaldehyde, embedded in paraffin, and cut into 5 μm sections. Alizarin blue was used to characterize the chondrocyte phenotype.

(3) Osteogenesis induction and characterization: Six-well plates were coated with 0.1% gelatin for 40 min in a ultra-clean hood and air dried. ADSC or MSC at passage 4 were inoculated in gelatin-coated plates to reach 60% confluency. For the induction, 2 mL of osteogenic induction medium was added to the wells, refreshed every 3 days for 3 weeks. For characterization of osteoblasts, the cells were washed with PBS, fixed with 4% neutral formaldehyde, stained in alizarin red staining solution for 5 min, and then rinsed for three times with PBS, and observed under microscope.

### 2.3. GFP Labeling of MSC, ADSC and Dil Labeling of CSVF

MSC and ADSC were stably transfected with lentivirus expressing GFP (GeneCopoeia, Rockville, MD, USA) to allow cell tracking after implantation. For the procedure, cells at passage 4 were cultured with antibiotic-free medium until 50% confluency, incubated with 2 μL of virus solution for 6 h with a virus titer of 10 in a 37 °C incubator, then changed to complete medium. The transfected cells were subjected to cell sorting for GFP by flow cytometry (BECKMAN COULTER, MoFlo Astrios EQs, USA). CSVF in 1 mL of culture medium was incubated with 2 μL of Dil reserve solution (Solarbio, Beijing, China) at 37 °C in dark for 5 min, and then incubated at 4 °C for 15 min in dark, centrifuged at 1500 rpm for 3 min, and washed twice with PBS. The result of the labelling was observed under a fluorescence microscope (Zeiss, Axio Observer3, Jena, Germany).

### 2.4. Establishment of ASI Rat Model

This study was approved by the Ethics Committee of the Medical School of South China University of Technology. Forty specific-pathogen free (SPF)-grade female SD rats at 6–8 weeks (200–230 g) were procured from the Experimental Animal Center of Southern Medical University. It has been shown that the type of anesthetic drug has an effect on the functional test results [42]. We used pentobarbital, which was used in the study by Peng Li et al. [14]. We adopted a modeling method with 25% anal sphincterectomy [43]. For the procedure, the animals were anesthetized, fixed, and disinfected. First, the surgical areas were marked, and then the ventral sphincter complexes of the rats were excised (about 1 cm × 0.5 cm). We resected 25% of the internal anal sphincter and external anal sphincter. After resection, the left and right sphincters were revealed, and the upper and lower cuts were made to the edges of the anuses and urethras. Buprenorphine was given for 2 days postoperatively for analgesia. The rats were divided into 5 groups, 8 animals for each group: healthy control group (8), PBS treatment group (8), MSC treatment group (8), ADSC treatment group (8), and CSVF treatment group (8). The healthy control group did not undergo surgery nor receive any intervention. The other groups received the injection of 0.2 mL of PBS, or 2 × 10^7^ cells/0.2 mL MSC, ADSC, or CSVF into both sides of the injury area (vertical anal margin side) marked at the time of mapping at 1 day after modeling, respectively. After injection, the small dermatome was allowed for 1 min to decrease in size before withdrawing the syringe needle. Half of the animals were assessed after 5 days, and the other half were assessed after 10 days of treatment to evaluate the degree of tissue repair and function recovery.

### 2.5. Functional Measurements

Function test includes anal pressure and electromyography measured in rats 1 day before and immediately after modeling and 5 and 10 days after treatments. The SD rats were anaesthetized by intraperitoneal injection of 2% pentobarbital at the dose of 0.3 mL/100 g body weight. The rat anal pressure test was performed with a homemade balloon catheter connected to a pressure transducer and a bioinformation acquisition system [14] (RM6240E bioinformation acquisition system, Chengdu Instrument Factory). The balloon catheter was emptied of air bubbles, lubricated with paraffin, and inserted into the rat’s anus about 0.5 cm deep. After the catheter was filled with 0.5 mL of saline, the bioinformation acquisition system was turned on to record for about 15 min after the waveform was stabilized. For the electromyography, three EMG needles were used for rat anal EMG detection, which were connected to the rats’ front paws and the anuses at 3-point and 9-point positions at a depth of about 4 mm. The EMG activity was recorded after waveform stabilization.

### 2.6. Immunohistochemical Staining

After the execution of animals with an overdose of anesthetic and images were taken of gross appearance of the anal area, muscle specimens were gently separated, fixed in 4% paraformaldehyde, and cut into 5 µm sections. Cryosections were also prepared for immunofluorescent staining. The sections underwent Masson Trichrome staining to distinguish collagen fibers, muscle fibers and connective tissues. Masson staining results were quantitatively calculated using ImageJ software. The cryosections were immune-stained for proliferating cell nuclear antigen (PCNA) to check the proliferation status of injected cells, for MyoD to check the differentiation of injected cells into myosatellite cells, and for α smooth muscle actin (α-SMA) to investigate the differentiation of injected cells into smooth muscle cells. For the immunohistochemical staining, after heat antigen retrieval, the slices were blocked in 10% BSA and incubated with primary antibodies, including anti-GFP antibody (PTG,50430-2-A), anti-α-SMA antibody (PTG,14395-1-A), anti-PCNA antibody (PTG,10205-2-A), and anti-MyoD antibody (PTG,18943-1-A), in a refrigerator at 4 °C overnight and washed three times with PBS. The secondary antibodies were carefully added and incubated in a 37 °C oven for 1 h. PBS rinsed. Cell nuclei was stained with 40,6-diamidino-2-phenylindole (DAPI). Slides were washed in PBS (pH = 7.4) in a decolorized shaker for 3 times, then sealed with anti-fluorescence quenching sealer and photographed under fluorescence microscope.

### 2.7. Data Analysis

All data was presented as mean ± standard deviation. In order to eliminate the influence of individual differences on the experimental results, the basal and peak anal pressure or peak EMG and frequency values of each rat before modeling was used as the baseline, and the data after modeling and after treatment was normalized to the baseline, which was first compared between groups. The changes of anal pressure basal and peak values, and EMG peak and frequency were analyzed by 2-way ANOVA using GraphPad Prism 9, followed by Bonferroni’s test. A *p* value < 0.05 was considered as a statistically significant difference.

## 3. Results

### 3.1. Characterization of MSC and ADSC

#### 3.1.1. Identification of MSC and ADSC Differentiation

**Figure 1:**
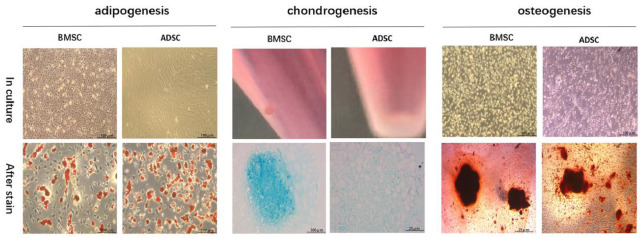
Differentiation of BMSC and ADSC into adipocytes, chondrocytes and osteoblasts. Representative images showed the cell morphology in culture after induction (top row) and the staining results of oil red stain for adipogenesis, alcian blue stain for chondrogenesis, and alizarin red stain for osteogenesis.

We first characterized the expanded MSC and ADSC by three lineage differentiation tests (Figure 1). MSC and ADSC showed positive staining for oil red after adipogenic induction. After chondrogenic induction, MSC and ADSC stained positive for alizarin blue. After osteogenic induction, MSC and ADSC stained positive for alizarin red. These results confirmed the differentiation capability of the expanded MSC and ADSC.

#### 3.1.2. Fluorescent Labeling of ADSC, MSC, and CSVF

**Figure 2:**
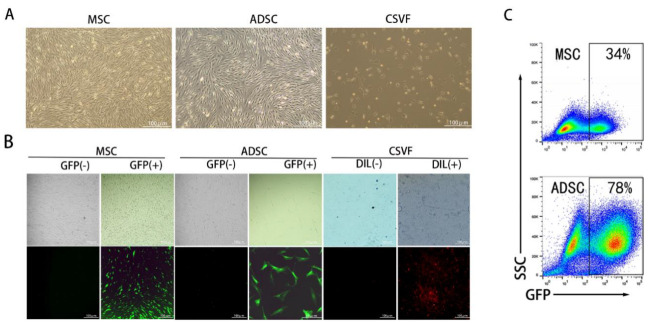
Cell morphology and fluorescent labeling of MSC, ADSC and CSVF. (**A**) Morphology of MSC and ADSC in culture and morphology of CSVF after extraction and change of culture medium. (**B**) The first row shows the phase contrast images of MSC, ADSC in the culture dish ready for sorting and the image of CSVF extracted from rat adipose tissue. The second row shows the fluorescence images of MSC, ADSC, and DIL-stained CSVF after transfection with lentivirus in order. (**C**) the flow cytometric analysis of MSC and ADSC.

Lentiviral long-lasting labeling was performed on ADSC and MSC. Based on the combined consideration of cell viability and GFP positivity after the labelling, a virus titer of 4 was determined for MSC, while for ADSC, it was 10. Microscopic observation confirmed that most of MSC and ADSC expressed GFP. The CSVF was successfully labeled with DIL and emitted red fluorescence (Figure 2B). Then the MSC and ADSC were analyzed to sort GFP positive cells using an ultra-high speed flow cell sorter. The successful labeling rate was 78% for ADSC and 34% for MSC (Figure 2C).

### 3.2. Functional Analysis of the Anal Sphincter

#### 3.2.1. Anal Sphincter Pressure Analysis

##### Anal Pressure Basal Values

No mortality or morbidity was noted after treatment during the course of this experiment.

**Figure 3:**
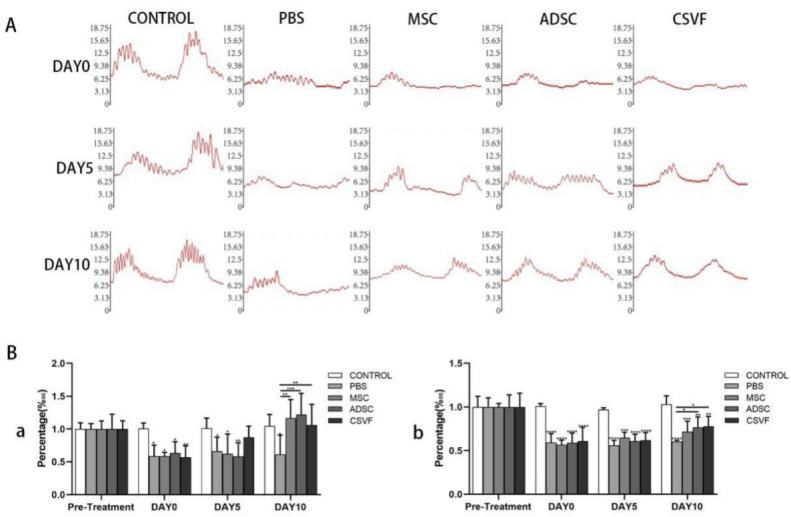
Anal pressure of control, PBS, MSC, ADSC and CSVF groups after modeling and after 5 and 10 days of treatment. (**A**) The first row shows the anal pressure graphs of 70 s recorded in control, PBS, MSC, ADSC and CSVF groups after modeling, and the anal pressure decreased significantly after modeling; the second row shows the anal pressure graphs of 70 s recorded in each group after 5 days of treatment; the third row also shows the anal pressure graphs of each group after 10 days of treatment. (**B**) Percentage of anal pressure basal value (a), anal pressure peak (b) in different treatment groups normalized to pre-treatment healthy controls. Data in each bar graph are expressed as mean ± standard deviation. An *, **, *** or **** without a horizontal line indicates a statistical difference in comparison to the control group, and an *, ** or *** with a horizontal line indicates a statistical difference between the groups at the horizontal ends. Significant differences between groups were expressed as **** *p* < 0.0001, *** *p* < 0.001, ** *p* < 0.01, and * *p* < 0.05.

The basal anal pressure values of the control, PBS, MSC, ADSC and CSVF groups were measured and presented in Figure 3 and Table 1. Before the anal sphincterectomy, there was no significant difference in the basal anal pressure values between the groups (Table 1). Immediately after surgery, the basal values decreased significantly in each group (PBS 4.10 ± 0.88; MSC 4.21 ± 0.33; ADSC 4.47 ± 01.00; CSVF 4.24 ± 1.12) compared to pre-surgery. At day 5 after treatment, the basal values of the PBS, MSC, and ADSC groups showed no significant recovery (PBS 4.70 ± 1.25; MSC 4.07 ± 1.61; ADSC 4.14 ± 1.28; CSVF5.29 ± 0.72) compared with day 0, and all the values were statistically lower than the control group on day 5 (*p* < 0.05).When the three cell implantation groups were compared, CSVF group had significantly higher effect than MSC and ADSC group. On day 10, the basal value of PBS treatment group (4.23 ± 1.65) was still significantly lower than the control group (*p* = 0.016), indicating limited self-repair. Compared to the PBS group, MSC, ADSC, and CSVF groups had significant higher basal values (*p* < 0.05). While the trend was higher for the treatment groups compared to control, the differences are not statistically significant. There was no statistical difference between the MSC, ADSC, and CSVF groups after 10 days of treatment, indicating a similar effect of these three groups.

##### Peak anal Pressure

Before the anal sphincterectomy, there was no statistical difference (*p* > 0.05) in the peak pressures measured in groups receiving treatments (control 14.94 ± 1.49; PBS 15.30 ± 1.31; MSC 15.95 ± 0.055; ADSC 15.31 ± 1.72; CSVF 16.33 ± 2.10). Immediately after the anal sphincterectomy, the peaks in the PBS, MSC, ADSC, and CSVF groups (PBS 9.06 ± 1.27; MSC 8.05 ± 1.17; ADSC 9.00 ± 1.17; CSVF 9.95 ± 2.21) significantly decreased compared to the control group (15.06 ± 0.37, *p* < 0.05). On day 5 after treatment, the peak pressure increased insignificantly in each treatment group compared to day 5 PBS group, but the values were significantly lower compared to the control group (*p* < 0.05). On day 10, there was a significant increase in pressure in each group compared to the data on day 0, but it was still significantly lower than the pre-modeling peak pressure (*p* < 0.05). Noteworthy, a significant increase in pressure was found in the ADSC group (11.71 ± 1.59) and the CSVF group (12.73 ± 2.45) compared to the PBS group (*p* < 0.05). There was no statistical difference in peak anal pressure between the MSC, ADSC, and CSVF groups on day 5 and day 10, indicating a similar effect in terms of peak anal pressure recovery, though the ADSC and CSVF groups behaved slightly better than the MSC group.

#### 3.2.2. EMG Analysis of the Anal Sphincter

##### Peak EMG

**Figure 4:**
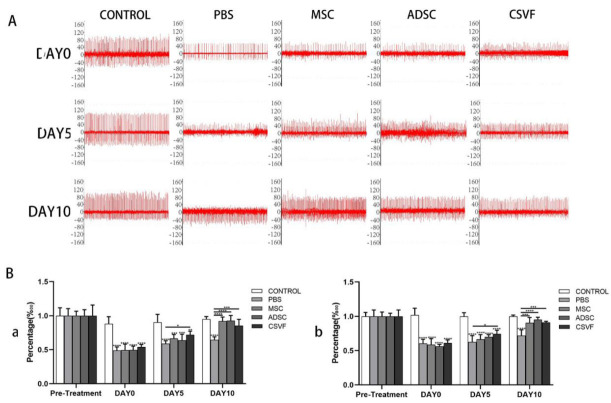
EMG of control, PBS, MSC, ADSC, CSVF groups after modeling and after 5 and 10 days of treatment. (**A**) The first row shows the EMG of control, PBS, MSC, ADC and CSVF groups after modeling, the second row shows the EMG of each group after 5 days of treatment, and the third row shows the EMG of each group after 10 days of treatment. (**B**) Percentage of EMG frequency (a), EMG peak (b) in different treatment groups normalized to pre-treatment healthy controls. Data in each bar graph are expressed as mean ± standard deviation. An **, *** or **** without a horizontal line indicates a statistical difference in comparison to the control group, and an *, *** or **** with a horizontal line indicates a statistical difference between the groups at the horizontal ends. Significant differences between groups were expressed as **** *p* < 0.0001, *** *p* < 0.001, ** *p* < 0.01, and * *p* < 0.05.

The EMG peaks (Figure 4 and Table 2) were basically the same without significant differences in all groups before surgery, and after surgery, the peak EMG in the treatment groups was significantly lower (PBS 84.89 ± 9.42; MSC 88.03 ± 7.07; ADSC 84.33 ± 2.28; CSVF 78.00 ± 3.58) than the control group (137.52 ± 6.93) (*p* < 0.001). At day 5, the peak EMG values increased in all groups (PBS 86.56 ± 9.46; MSC 93.53 ± 8.24; ADSC 100.87 ± 3.99; CSVF 104.02 ± 6.34) compared to day 0, though not significantly, and the CSVF group had significantly more peak increases than the PBS group (*p* = 0.015), while the ADSC and MSC groups showed no such significance. At day 10, the peak EMG values increased significantly in the MSC, ADSC and CSVF groups (MSC 128.87 ± 9.60; ADSC 130.64 ± 3.64; CSVF 124.69 ± 1.99) compared with the PBS group (95.16 ± 9.81), indicating enhanced repair by injected cells. The peaks of the MSC, ADSC, and CSVF groups were basically close to the pre-modeling level, and no significant difference was found between the MSC, ADSC, and CSVF groups.

##### EMG Frequency

There was no statistical difference in the EMG frequency (Figure 4 and Table 2) between groups before modeling. Modeling led to a significant decrease in EMG frequency in the PBS, MSC, ADSC, and CSVF groups (PBS 15.33 ± 2.05; MSC 14.33 ± 1.69; ADSC 14.33 ± 3.39; CSVF 14.66 ± 0.47) compared with control group (26.00 ± 0.81) (*p* < 0.001), and the frequency of each group increased with no significant difference after 5 days of treatment (PBS 17.25 ± 1.08; MSC 19.25 ± 1.47; ADSC 19.00 ± 2.16; CSVF 20.66 ± 1.24). There was no statistically significant difference between the three groups of MSC, ADSC, and CSVF compared with the PBS group. After 10 days of treatment, the EMG frequencies in the MSC, ADSC, and CSVF groups (MSC 25.75 ± 1.47; ADSC 25.66 ± 1.69; CSVF 22.25 ± 2.04) increased significantly (*p* < 0.05) compared to the PBS group (18.25 ± 1.08) and were almost comparable to the control group (26.00 ± 0.81). However, no statistical difference was found between the MSC, ADSC, and CSVF groups after 5 days of treatment and after 10 days of treatment (*p* < 0.05), indicating a similar effect on the elevation of EMG frequency.

### 3.3. Gross Healing of the Surgical Sites

**Figure 5:**
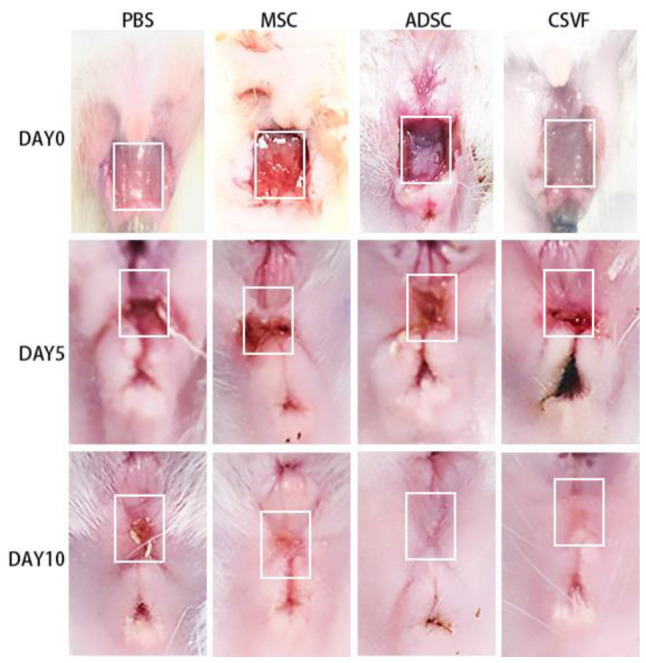
Wound healing in the PBS, MSC, ADSC and CSVF groups on day 0, 5 and 10 after treatment. The first row shows the pictures of the incision in each group of PBS, MSC, ADSC, and CSVF after modeling, the second row shows the healing after 5 days of treatment; the third row shows the healing of the incision after 10 days of treatment. The white box in the figure indicates the injury site.

Figure 5 showed the gross appearance of the wound on days 0, 5, and 10 after surgery. On day 5, the wounds were close to healing, but there were still blood scabs, and the wounds in each group showed obvious inward contraction. On day 10, the wounds in all groups appeared healed, except for the PBS group, but the wounds were not completely filled with tissue and had obvious depression.

### 3.4. Masson Trichrome Stain

**Figure 6:**
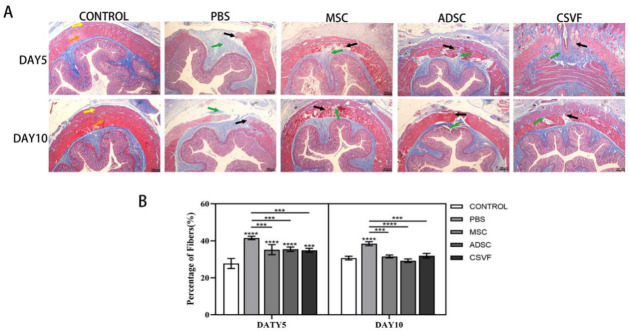
(**A**) Masson Trichrome staining of the injury site in the control group, PBS group, MSC group, ADSC group and CSVF group after 5 and 10 days of treatment. Yellow and orange arrows represent the external anal sphincter and internal anal sphincter, respectively, green arrows point to regenerated connective tissue, while black arrows point to regenerated muscle. (**B**) Percentage of fibers in the control group, PBS group, MSC group, ADSC group, and CSVF group after 5 and 10 days of treatment. An **, *** or **** without a horizontal line indicates a statistical difference in comparison to the control group, and an **, *** or **** with a horizontal line indicates a statistical difference between the groups at the horizontal ends. Significant differences between groups were expressed as **** *p* < 0.0001, *** *p* < 0.001, ** *p* < 0.01, and * *p* < 0.05.

The anal specimens were then subjected to Masson Trichrome staining. As shown in Figure 6, on day 5 after treatment, the PBS group still had a large wound, while the MSC group had a smaller wound, and the ADSC and CSVF groups were close to healing. On day 10, the PBS group had a small amount of new muscle covering part of the wound, while the MSC group was close to healing, and the muscle in the CSVF and ADSC groups healed better than those on day 5. Based on our observation, muscle accounted for significantly more of the new tissues than fibers did, while connective tissue accounted for even less. Quantification of fibers in each group also revealed that the MSC, ADSC, and CSVF groups had significantly lower proportions of fibers than the PBS group.

### 3.5. The Survival, Proliferation and Differentiation of MSC, ADSC, and CSVF In Vivo

**Figure 7:**
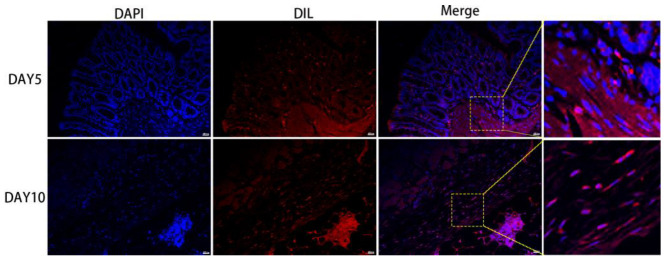
Fluorescence of DIL labeling after 5 and 10 days of treatment in CSVF group. Cell nucleus was stained in blue with DAPI, and DIL-labeled CSVF was visualized by red fluorescence.

**Figure 8:**
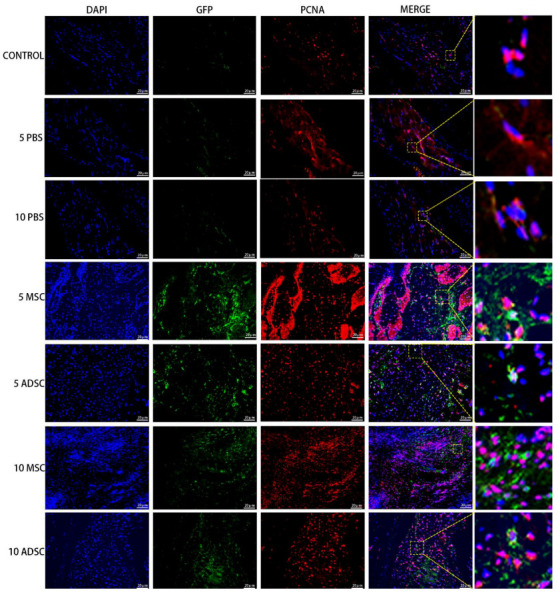
ADSC and MSC co-expressed GFP and PCNA after 5 and 10 days of injection. The 1st to 7th row represents staining of GFP and PCNA in the control group, PBS group on the 5th and 10th day, MSC and ADSC group on the 5th day, and MSC and ADSC on the 10th day, after the treatment.

**Figure 9:**
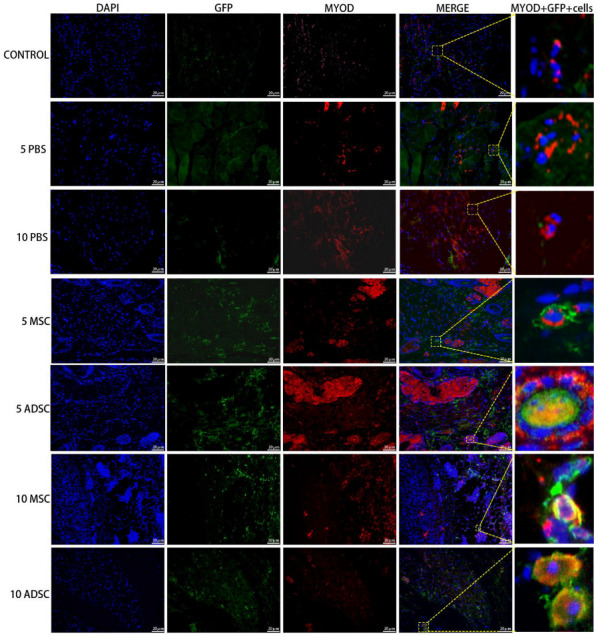
ADSC and MSC co-expressed GFP and MYOD after 5 and 10 days of injection. The 1st to 7th row represents staining of GFP and MyoD in the control group, PBS group on the 5th and 10th day, MSC and ADSC group on the 5th day, and MSC and ADSC on the 10th day, after the treatment.

**Figure 10:**
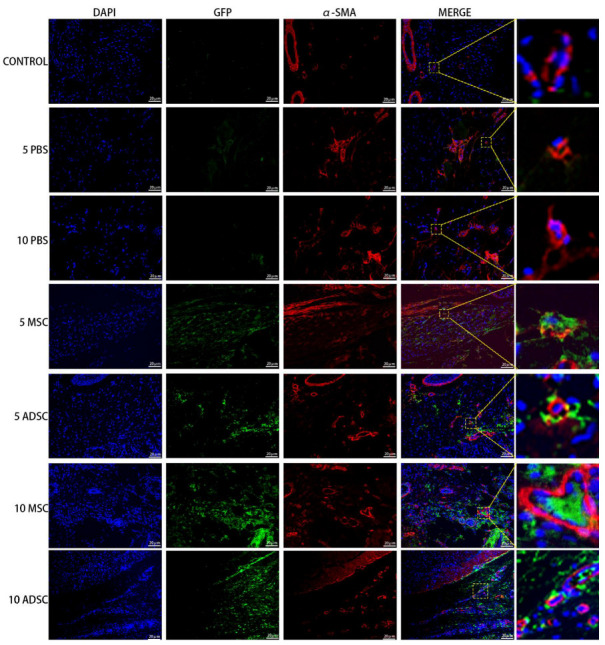
ADSC and MSC co-expressed GFP and α-SMA after 5 and 10 days of injection. The 1st to 7th row represents staining of GFP and α-SMA in the control group, PBS group on the 5th and 10th day, MSC and ADSC group on the 5th day, and MSC and ADSC on the 10th day, after the treatment.

The anal tissues of the rats were then further analyzed by immunofluorescent staining to track the survival, proliferation, and differentiation of GFP positive MSC and ADSC, or DIL-labeled CSVF. The survival was tracked by green fluorescence of GFP or red fluorescence of DIL, and the proliferation was visualized by the co-expression of PCNA, proliferation marker. As shown in Figure 7 and Figure 8, GFP+ ADSC and MSC as well as DIL+ CSVF were found in vivo after 5 and 10 days of treatment. Some of the GFP+ MSC were found to express PCNA on day 5 and day 10, indicating the proliferation of implanted MSC. Similarly, the co-expression of GFP and PCNA was also found after 5 and 10 days of ADSC treatment, indicating that the injected ADSC were still in a proliferative state till day 10. The differentiation of the implanted cells into myosatellite cells was investigated by the expression of MyoD, and the differentiation of the implanted cells into smooth muscle cells was investigated by the expression of α-SMA. After 5 and 10 days of treatment, some of the GFP+ MSC and ADSC co-expressed MyoD (Figure 9), indicating differentiation to myosatellite cells by MSC and ADSC. Meanwhile, α-SMA was found to be expressed by some of the GFP+ MSC and ADSC after 5 and 10 days of treatment (Figure 10), indicating that the implanted MSC and ADSC have given rise to smooth muscle cells.

## 4. Discussion

In the literature, several animal models of anal sphincter injuries have been reported, such as anal sphincterectomy [43], 25% anal sphincterectomy [44], 50% anal sphincterectomy [45], cryoinjury [46], nerve injury [47], etc. There are also choices of resection sites, as some choose to resect the posterior sphincter [48] while others resect the left semicircle [15], etc. This leads to poor homogeneity between studies and makes the re-analysis of data in different studies difficult. In our study, on day 5, the incisions in the PBS injection group were close to healing. Comparing with previous 25% anal sphincterectomy studies by others at adjacent time points [14,17,44], it appeared that the rats in our modeling showed faster healing and tissue sectioning. This may first be due to differences in observation time. We used the 5th and 10th day after mold to observe the healing, while others used observation times of 4 days, 7 days, 14 days, etc. The inconsistent observation times may be responsible for some differences. However, we think the method of modeling and sample embedding may be the most important influencing factors. We chose the modeling method of ventral 25% anal sphincter excision, which includes the entire tissue between the ventral internal anal sphincter and the outer skin layer, which may result in wound contraction and close proximity of the wound ends without complete reliance on regenerative repair of the injury. The best modeling method would be to remove only the muscle layer without the outer skin layer when 25% of the anal sphincter complex is excised, i.e., by making a small incision at the anal verge and separating the anal sphincter complex inward, but the operation would be difficult. Another influencing factor is the way of sample harvest. Most studies chose to completely peel the skin outside the anal sphincter complex, which can lead to a change in the relative position of the muscles at the wound end. We kept the skin at the healing site and peeled the skin at the other parts, which can remain the original position of the injury site as much as possible.

The current stem cell delivery methods used in the treatment of fecal incontinence include cell sheet and biosutures in addition to cell injection. In a study by Yusuke Inoue et al. [15], temperature-responsive culture dishes were used to prepare ADSC sheets, which were transplanted to anal sphincter defective site and significantly improved anal pressure in rats with anal dysfunction. Trébol et al. [42] prepared biosutures by culturing 30 cm 6/0 polyglactin 910 sutures with 1.5 × 10^6^ ADSC in ultra-low attachment plates. They found that the number of cells on the sutures decreased after two stitches through muscle tissue, so each biosuture was used only twice. They also found that the cells in the injection group formed a “conglomerate” structure, while the cells in the biosutures group attached to the suture and migrated towards the injury site. For cell injection, cells can be delivered via intramuscular or intravenous approach [14].

Studies of MSC on anal sphincter injury have been carried on rats [44], rabbits [13] and other animals. Currently, the repair of anal sphincter injury by MSC or ADSC [15] has been independently reported. Peng Li et al. [14] compared the therapeutic effects of intramuscular and intravenous injection of MSC. 10^7^ MSC were injected once a day and repeated for 7 days. After 14 and 28 days of treatment, the peak anal pressure contraction, peak EMG, and frequency improved more significantly in the intramuscular injection group than intravenous injection group, and the percentage of new muscle was higher in the intramuscular injection group. Salcedo et al. selected 24 h after resection of 25% of the anal sphincter as the time point to inject MSC, as their previous studies showed a substantial increase in the expression of cytokines such as SDF-1 after 24 h of injury [13]. They also injected 0.2 mL PBS into the anal sphincter. They used a MSC cell number of 5 × 10^5^, while other studies have used 2 × 10^6^ [12]. Considering possible cell death after implantation, we chose the cell number of 1 × 10^7^ for our study. Inoue et al. [15] found that the transplantation of ADSC sheets into the injured area brought a significant improvement on anal pressure.

Currently, it is yet unclear how the efficacy of ADSC compared to MSC in treating anal sphincter injury. In our study, we found that ADSC was slightly more effective in histological recovery than MSC in repairing ASI, and the functional test showed that ADSC had a slightly higher, though not significant, effect compared to MSC in promoting the recovery of muscle contraction and myoelectric activity. In summary, the efficacy of ADSC is close to, if not better, than MSC; therefore, ADSC can be an ideal alternative therapeutic cell source for repairing ASI.

In recent years, many studies have explored therapeutic effects between ADSC and SVF on other diseases, including osteochondral defects [49], encephalitis [50], etc. They found that SVF has similar or even better therapeutic effects to ADSC in treating these diseases. In our study, CSVF also showed a significant increase in the basal and peak anal pressure values, and the frequency and peak EMG, on day 5 compared to ADSC and MSC. However, on day 10, except for the peak anal pressure, which was slightly higher than ADSC and MSC, all other functional indexes were lower than ADSC and MSC. We speculate that the reason may be due to the stem cell number in CSVF is less than ADSC and MSC. Since we injected the same number of cells in each group, and the stem cell rate in CSVF is only 9.5% [51], the CSVF group received a much lower amount of stem cells compared to ADSC and MSC. Therefore, though CSVF may have gained the lead in the short run, they fell behind due to their lower stem cell content while ADSC and MSC engrafted and showed a long-term effect.

It is worthy to mention that recently some clinical studies have investigated the safety and efficacy of stem cells to treat anal sphincter incontinence. Most of these studies used autologous myoblasts (AM) [18,19,52,53,54,55]. AM injection significantly improved median Cleveland Clinic Incontinence (CCI), Wexner scores, Fecal Incontinence Quality of Life (FIQL) scores, and patient conditions when assessed at 12 months [49,50] or up to 5 years [54,55] after the injection. A few studies have also investigated the effect of ADSC in human subjects. Sarveazad et al. [56] published a randomized controlled trial that included 20 patients with fecal incontinence. Patients were injected with 6 × 10^6^ ADSCs or PBS in the experimental and control groups, respectively, after the implementation of non-overlapping sphincteroplasty. After two months, there was no significant difference in the Wexner scores, but the ADSCs-treated group showed a significant increase in EMG activity and lesion area of muscle ratio. de la Portilla et al. [57] included a total of 16 patients with FI treated with autologous ADSC and showed that this treatment was safe, but they failed to demonstrate significant efficacy to improve the patient situations. Currently, there are no clinical trials using MSC and cSVF to treat FI.

The process of repairing the anal sphincter after injury has not been adequately studied. It is possible that the mechanism is similar to other sphincter injury repairs [58], where new smooth muscle and collagen fibers are produced by myosatellite cells [59] or fibroblasts. It is believed that stem cells are recruited to migrate towards the site of injury by cytokines such as SDF-1 [60,61]. Our immunohistochemical results showed that the injected stem cells remained alive for 5 and 10 days post injection, and a small number of cells expressed myosatellite cell marker, suggesting that the injected stem cells could differentiate into myosatellite cells, which in turn differentiate into myocytes to repair the anal sphincter injury. Besides giving rise to myosatellite cells, the therapeutic effects of MSC, ADSC, and CSVF may also be delivered by paracrine effect by secretion of cytokines to promote local cell activities, which requires further investigation. Meanwhile, the time points selected for post-treatment sampling and functional testing in this study were 5 and 10 days, with the aim of assessing the short-term efficacy of MSC, ADSC, and CSVF on ASI. The results showed that the anal sphincter function was not fully restored, so an investigation of a longer time point may be helpful to validate the efficacy of the three therapies on ASI. Meanwhile, we observed an interesting result that after 10 days of treatment, the basal anal pressure values in the MSC, ADSC, and CSVF groups had all reached normal values, but the peak anal pressure values were still lower than normal. From the analysis of the experimental results, the possible reason for this result may be that after 10 days of treatment, the damaged part of the anus has healed (Figure 5) and formed a complete annulus, which led to a subsequent increase in the basal anal pressure values. However, the internal muscles and the nerves innervating them had not fully recovered and failed to establish the same strong contractions as in normal conditions.

The mechanisms of MSC, ADSC, and CSVF in the treatment of fecal incontinence remain incompletely understood. The multidirectional differentiation potential, immunomodulation, secretory function, promotion of angiogenesis, and the matrix components contained in CSVF may play an important role in therapy. Studies have shown [62] that the differentiation ability of MSC with great potential was mainly verified by in vitro experiments, while in vivo experiments showed mainly protective effects. In this experiment, MSC and ADSC were transplanted and differentiated into myosatellite cells, and some of the experimental results showed that they also differentiated into smooth muscle cells. We need to further explore whether similar phenomenon occurred in CSVF. Meanwhile, MSC can suppress inflammation and reduce tissue fibrosis [63]. Stem cells may also participate in immune responses via multiple pathways; for example, MSC can regulate immune responses through paracrine secretion, cell-cell contact, and extracellular vesicles [64,65]. Moreover, stem cells can interact with the local microenvironment to influence both the function of cells and the component of microenvironments [66,67]. MSCs and ADSCs can stimulate neighboring tissue cells, and Nakamura et al. [68] found that MSCs can secrete exosomes to promote muscle regeneration by promoting muscle and angiogenesis. The mechanism by which CSVF exerts its therapeutic effects may be similar to that of ADSC but may be more complex because it contains cellular components other than ADSC.

No adverse effects associated with the intervention were observed in our study, which is consistent with most current reports that stem cell therapy is safe and well tolerated. However, occasional adverse events have been reported. Jacobs et al. [16] found that though no distant metastases of stem cells was found in the liver or lungs, two SD rats developed abnormal growth foci (tumors) in the anal sphincter. Ajay E. [69] reported three women suffering from macular degeneration after undergoing SVF therapies. They developed complications including vision loss, detached retinas, and bleeding and are now totally blind. There is another report of bilateral retinal detachment after SVF injection.

Our study has some limitations. The culture, labeling, and sorting process of stem cells resulted in a P7 generation of stem cells used for treatment, which affects the therapeutic effect while their multidirectional potential decreases with passaging. Using stem cells of earlier passage may exhibit more satisfied therapeutic effects. Secondly, we have only investigated the effect of stem cells up to 10 days after the injection. A longer time point may help to validate the long-term effects of stem cells in treating ASI. The molecular mechanisms, such as the paracrine effects, have not been fully investigated, which is something we plan to explore in the future.

## 5. Conclusions

MSC, ADSC, and CSVF showed promising effects on acute anal sphincter injury. In the early stage (day 5) of repair, CSVF showed highest therapeutic effect, while as time lengthened (day 10), MSC and ADSC showed a higher therapeutic effect than CSVF. When comparing the effects of MSC and ADSC, ADSC was slightly better than MSC in the indexes of anal pressure, etc. A large number of MSC, ADSC, and CSVF were still alive in vivo after 10 days of injection, and a small subset of injected stem cells have differentiated into myosatellite cells. Overall, ADSC and CSVF are suitable alternative cell sources for ASI repair.

## Figures and Tables

**Table 1:** Mean ± standard deviation of anal pressure basal and peak values before modeling and after 5 days and 10 days of treatment in Control, PBS, MSC, ADSC, and CSVF groups.

Variable	Control	PBS	MSC	ADSC	CSVF
Baseline pressures (cmH_2_O) Pre-excision	7.47 ± 0.57	6.90 ± 0.44	7.18 ± 0.77	7.06 ± 1.30	7.42 ± 0.76
DAY(0)	6.88 ± 0.45	4.10 ± 0.88 *	4.21 ± 0.33 *	4.47 ± 1.00 *	4.24 ± 1.12 *
DAY(5)	7.22 ± 0.91	4.70 ± 1.25 *	4.07 ± 1.61 **	4.14 ± 1.28 **	5.29 ± 0.72
DAY(10)	7.15 ± 0.96	4.23 ± 1.65 *	8.43 ± 1.61 ^&&&^	7.4 ± 1.70 ^&&^	7.89 ± 2.10 ^&&^
Peak pressures (cmH_2_O) Pre-excision	14.94 ± 1.49	15.30 ± 1.31	15.95 ± 0.55	15.31 ± 1.72	16.33 ± 2.10
DAY(0)	15.06 ± 0.37	9.06 ± 1.27 ***	8.05 ± 1.17 ****	9.00 ± 1.17 ***	9.95 ± 2.21 ***
DAY(5)	15.61 ± 0.25	8.94 ± 0.75 ****	9.98 ± 0.79 ***	9.49 ± 1.07 ****	11.4 ± 2.08 **
DAY(10)	16.08 ± 1.22	9.64 ± 0.17 ****	11.45 ± 1.56 **	11.71 ± 1.59 **	12.73 ± 2.45 *

Significant differences compared to the control group were expressed as **** *p* < 0.0001, *** *p* < 0.001, ** *p* < 0.01, and * *p* < 0.05; Significant differences compared to PBS are expressed as ^&&&^ < 0.001 and ^&&^ < 0.01.

**Table 2:** Peak and frequency EMG plots of the control group, PBS group, MSC group, ADSC group and CSVF group.

Variable	Control	PBS	MSC	ADSC	CSVF
Peak EMG(μv) Pre-excision	134.65 ± 9.10	132.55 ± 6.55	142.40 ± 9.59	136.96 ± 4.53	137.00 ± 7.17
DAY(0)	137.52 ± 6.93	84.89 ± 9.42 ****	88.03 ± 7.07 ****	84.33 ± 2.28 ****	78.00 ± 3.58 ****
DAY(5)	142.44 ± 6.15	86.56 ± 9.46 ****	93.53 ± 8.24 ****	100.87 ± 3.99 ****^&^	104.02 ± 6.34 ****^&^
DAY(10)	140.09 ± 2.12	95.16 ± 9.81 ****	128.87 ± 9.60 ^&&&&^	130.64 ± 3.64 ^&&&&^	124.69 ± 1.99 *^&&&&^
EMG frequency(Hz) Pre-excision	27.33 ± 1.24	28.33 ± 2.86	28.00 ± 2.94	27.66 ± 2.62	26.00 ± 2.73
DAY(0)	26.00 ± 0.81	15.33 ± 2.05 ****	14.33 ± 1.69 ****	14.33 ± 3.39 ****	14.66 ± 0.47 ****
DAY(5)	28.00 ± 2.94	17.25 ± 1.08 ****	19.25 ± 1.47 ****	19.00 ± 2.16 ****	20.66 ± 1.24 ***
DAY(10)	26.00 ± 0.81	18.25 ± 1.08 ***	25.75 ± 1.47 ^&&&&^	25.66 ± 1.69 ^&&&^	22.25 ± 2.04 *^&^

Significant differences compared to the control group were expressed as **** *p* < 0.0001, *** *p* < 0.001, and * *p* < 0.05; Significant differences compared to PBS are expressed as ^&&&&^ < 0.0001, ^&&&^ < 0.001 and ^&^ *p* < 0.05.

## Data Availability

The data presented in this study are available on request from the corresponding author.
